# Correlation between estrogen receptor expression and prognosis in epithelial ovarian cancer: a meta-analysis

**DOI:** 10.18632/oncotarget.18253

**Published:** 2017-05-29

**Authors:** Zhaojun Shen, Hui Luo, Saisai Li, Bo Sheng, Menghuang Zhao, Haiyan Zhu, Xueqiong Zhu

**Affiliations:** ^1^ Department of Obstetrics and Gynecology, The Second Affiliated Hospital of Wenzhou Medical University, Wenzhou 325027, Zhejiang Province, China

**Keywords:** epithelial ovarian cancer, estrogen receptor, prognosis, meta-analysis

## Abstract

**Objective:**

Accumulated studies have investigated the prognostic significance of estrogen receptor expression in epithelial ovarian cancer, but results remain controversial. The aim of this study was to perform a meta-analysis to clarify the prognostic value of estrogen receptor expression in epithelial ovarian cancer.

**Methods:**

A systematic search was performed in PUBMED, EMBASE, and COCHRANE databases to identify relevant studies up to December 2016. The pooled hazard rates (HR) with 95% confidence intervals (CIs) for overall survival and time to tumor progression were calculated and then weighted and pooled in this meta-analysis with a random-effect model.

**Results:**

Thirty-five studies with a total of 5824 patients were included. In brief, the expression of estrogen receptor was associated with an improved overall survival (HR = 0.86, 95% CI = 0.76-0.97), whereas there was no significant difference between estrogen receptor and time to tumor progression among epithelial ovarian cancer patients. Subgroup analysis revealed that estrogen receptor expression was significantly correlated with overall survival in different subgroups, such as in unclassified epithelial ovarian cancer (HR= 0.80, 95% CI = 0.66-0.95), studies using immunohistochemistry detection method (HR= 0.85, 95% CI = 0.73-1.00), European population (HR= 0.75, 95% CI = 0.60-0.94) and estrogen receptor α subtype (HR= 0.78, 95% CI = 0.62-0.98).

**Conclusions:**

Estrogen receptor, especially estrogen receptor α, was associated with an improved overall survival in epithelial ovarian cancer. Estrogen receptor expression may be a promising prognostic factor in epithelial ovarian cancer patients.

## INTRODUCTION

Ovarian cancer is the second most common and leading lethal gynecological cancer [1]. About 90% of these subtypes are epithelial ovarian cancer (EOC). In the United States, approximately 22,440 new cases and 14,080 deaths from ovarian cancer were estimated in 2017 [1]. Despite considerable efforts have been made to improve surgical techniques and meticulously designed chemotherapy regimens, the 5-year survival rate remains 10% ∼ 30% [2–4]. The high rate of lethality and poor rate of survival are primarily due to late detection and rapid progression [2–4]. For these reasons, identifying reliable predictive biomarkers for prognosis and developing novel therapeutic strategies are urgently needed.

Estrogen and estrogen receptor (ER) have been well documented to be associated with ovarian cancer [5]. Estrogens promote physiological actions, such as cell survival and proliferation, after binding to their estrogen receptors (ERs) subtypes (ER α and ER β) [6]. The frequency of ER expression in human epithelial ovarian carcinoma has been reported varying in the range of 43%∼81% immunohistochemically [7, 8]. In ER-positive human ovarian cancers, estrogen promoted cancer cell growth *in vitro* [9]. Conversely, anti-estrogens inhibited cell growth both *in vitro* and *in vivo* [10].

Given its important role in ovary carcinogenesis, multiple studies have investigated the relationship between estrogen receptor expression and epithelial ovarian cancer clinical outcomes, with contradictory findings [9–13]. While researches by Bizzi et al [11] and Yang et al [12] reported that ER expression predicted an improved outcomes in epithelial ovarian cancer, Liew et al [4] reported that the expression of ER had no effect on clinical outcomes among epithelial ovarian cancer patients. On the contrary, Khandakar et al [14] supported a negative relationship between ER expression and overall survival of epithelial ovarian cancer. Thus, in epithelial ovarian cancers, the prognostic significance of estrogen receptor remains unclear.

A similar situation, the prognostic value of ER α and ER β in epithelial ovarian cancer patients was also controversial. While the expression of ER α was shown to predict a better prognosis in the research by de Toledo et al [15], Zamagni et al [16] reported a positive ER α status was associated with a negative prognosis of epithelial ovarian cancer in their study. The prognostic value of ER β in epithelial ovarian cancer patients also was controversial [3, 6]. Therefore, it was necessary to evaluate the association between ER expression and the survival of women with epithelial ovarian cancer by a meta-analysis.

In this study, we performed a meta-analysis to evaluate the prognostic value of ER and its two subtypes (ER α and ER β) in patients with epithelial ovarian cancer, aiming to provide more strategies for follow-up and targeted regimens.

## RESULTS

### Literature search results

**Figure 1:**
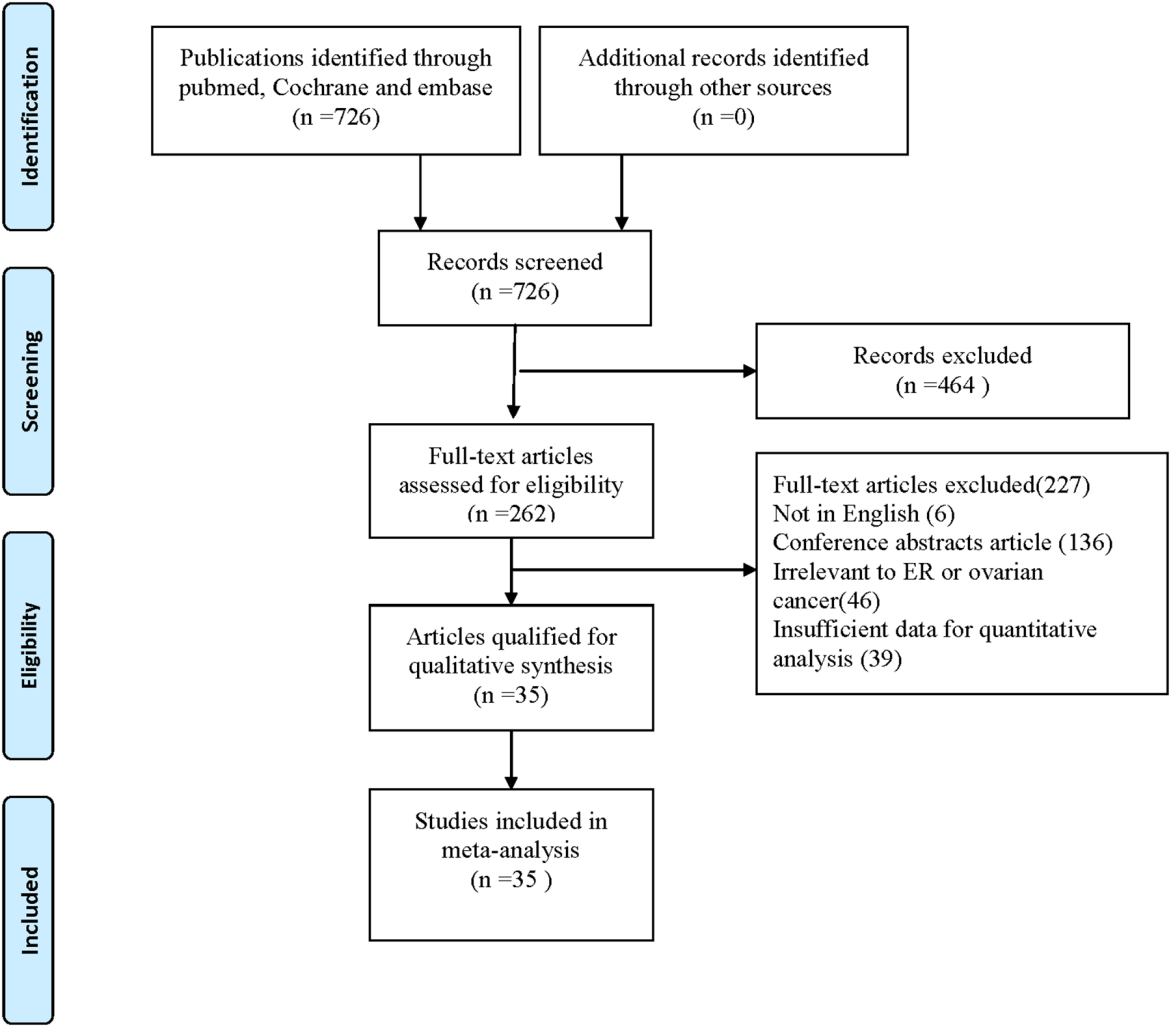
Flow of study identification, inclusion, and exclusion

Initially, 726 relevant citations were retrieved in PUBMED, EMBASE, and COCHRANE databases. After reviewing titles and abstracts, 464 studies were eliminated for clearly irrelevant. Among the remaining 262 studies, 6 studies were further excluded because they were not written in English; 136 articles were excluded because of conference abstracts or other studies; 46 studies were irrelevant to ovarian cancer and prognosis; 39 studies were excluded due to insufficient data for quantitative analysis. Ultimately, 35 studies [3–8, 11–39] with a total of 5824 patients were included in this meta-analysis. Details of the study selection process were presented in Figure 1.

### Characteristics of included studies

In this meta-analysis, while 35 studies described the correlation between overall survival (OS) and ER expression and 18 trials involved in disease-free survival (DFS), progression-free survival (PFS), and recurrence-free survival (RFS). Since DFS, PFS and RFS were similar in meaning, these three outcome endpoints were combined as an unified prognostic parameter, time to tumor progression (TTP) [40]. All of the selected studies focused on epithelial ovarian cancer, in which only 12 studies concentrated on serous ovarian cancer and the remaining 23 studies involved in various subtypes of epithelial ovarian cancers (unclassified epithelial ovarian cancer). Among 35 studies, 11 reporters evaluated the association between ER α and epithelial ovarian cancer clinical outcome and 8 studies investigated the prognostic value of ER β among epithelial ovarian cancer patients.

As for the region, 19 studies were performed in Europe, 7 studies in North America, 5 studies in Asia, 2 studies in South America, 1 study in Oceania, and 1 study was conducted across regions. With respect to detection method, 20 studies used immunohistochemical staining for estrogen receptor assessment, 2 studies used polymerase chain reaction, and the other 4 studies used dextran-coated charcoal method.

The quality of included studies was assessed by the Newcastle-Ottawa Scale (NOS) and the scores of all studies were more than six, suggesting a high methodological quality across all studies. Detailed characteristics of eligible studies were presented in Table 1.

**Table 1:** Characteristics of eligible studies

Study & year	Country	Region	Sample size(n)	Age (year)	Follow-up (months)	Way of evaluation	Pathological type	Method for data collection	Out comes	HR (95%CI)	NOS score
Jönsson 2015[3]	Sweden	Europe	118	Mean(range) 58(26-83)	60	IHC	EOC	Directly	OS	1.13 (0.78-1.64)	6
Jönsson 2015[3]	Sweden	Europe	118	Mean(range) 58(26-83)	60	IHC	EOC	Directly	PFS	1.20 (0.77-1.63)	7
Liew 2015[4]	China	Asia	108	Median 53	Median 41	IHC	EOC	Directly	OS	1.08 (0.85-1.36)	7
Liew 2015[4]	China	Asia	108	Median 53	Median 41	IHC	EOC	Directly	DFS	1.025 (0.81-1.30)	7
de Toledo 2014[15]	Brazil	South America	152	Mean(SD) 55.2(12.3)	Mean 43.6	IHC	EOC	Directly	OS	0.46 (0.22-0.95)	8
de Toledo 2014[15]	Brazil	South America	152	Mean(SD) 55.2(12.3)	Mean 43.6	IHC	EOC	Directly	DFS	0.35 (0.03-0.68)	7
Tkalia 2014[5]	Ukraine	Europe	232	Mean(SD) 51.7(0.8)	Mean(SD) 39.5(1.7)	IHC	EOC (serous)	Indirectly	OS	0.89 (0.64-1.23)	6
Tkalia 2014[5]	Ukraine	Europe	232	Mean(SD) 51.7(0.8)	Mean(SD) 39.5(1.7)	IHC	EOC (serous)	Indirectly	RFS	0.95 (0.68-1.33)	8
Ciucci 2014[6]	Italy	Europe	56	Median(range) 54 (33–79)	Mean(range) 47(9-162)	IHC	EOC (serous)	Indirectly	OS	2.8 (1.3-5.7)	8
Ciucci 2014[6]	Italy	Europe	56	Median(range) 54 (33–79)	Mean(range) 47(9-162)	IHC	EOC (serous)	Indirectly	DFS	1.6 (0.9-2.9)	7
Lenhard 2012[22]	Germany	Europe	155	Mean(range) 59 (21–88)	Median 146.4	IHC	EOC	Directly	OS	0.86 (0.52-1.43)	8
Høgdall 2007[8]	Denmark	Europe	580	NA	120	IHC	EOC	Directly	OS	0.8 (0.63-0.99)	6
García-Velasco 2008[13]	Spain	Europe	72	Median(range) 57(28-82)	Median(range) 33(1-193)	IHC	EOC	Directly	OS	0.23 (0.14-0.55)	7
García-Velasco 2008[13]	Spain	Europe	72	Median(range) 57(28-82)	Median(range) 33(1-193)	IHC	EOC	Directly	PFS	1.4 (0.73-2.93)	7
Bizzi 1988[11]	Italy	Europe	97	Median(range) 58(24-81)	36	DCCM	EOC	Directly	OS	0.4 (0.23-0.71)	7
Scambia 1995[26]	Italy	Europe	117	Range (40-60)	Median(range) 19(2-110)	DCCM	EOC	Indirectly	OS	0.92 (0.52-1.61)	8
Scambia 1995[26]	Italy	Europe	117	Range (40-60)	Median(range) 19(2-110)	DCCM	EOC	Indirectly	PFS	0.98 (0.58-1.66)	8
Kieback 1993[27]	USA	North America	42	NA	96	IHC	EOC (serous)	Indirectly	OS	0.72 (0.34-1.51)	7
Geisler 1996[28]	USA	North America	96	Mean(range) 59(38-88)	60	DCCM	EOC (serous)	Indirectly	OS	0.87 (0.51-1.48)	7
Athanassiadou 1998[29]	Greece	Europe	100	Mean(SD) 51.56(10.2)	28.5	DCCM	EOC	Directly	OS	0.89 (0.52-1.53)	6
Lee 2005[30]	USA	North America	322	Mean(range) 58.3(20-86)	Mean(range) 64(1-120)	IHC	EOC	Directly	OS	1.2 (0.8-1.8)	6
De Sousa Damião 2007[31]	Brazil	South America	40	Mean(range) 55.8(20-87)	120	IHC	EOC	Indirectly	OS	0.57 (0.3-1.09)	6
Yang 2008[12]	China	Asia	86	Median(range) 34.2(17-40)	60	IHC	EOC	Directly	OS	0.49 (0.19-1.25)	8
Buchynska 2009[32]	Ukraine	Europe	81	Mean(SD) 46.6(2.4)	60	IHC	EOC (serous)	Indirectly	OS	0.28 (0.14-0.54)	8
Arias-Pulido 2009[33]	USA	North America	134	Mean(SD) 54.1(14.3)	60	IHC	EOC	Indirectly	OS	1.02 (0.69-1.49)	7
Burges 2010[17]	Germany	Europe	100	Mean(range) 60.35(33.12-89.2)	160	IHC	EOC (serous)	Directly	OS	0.55 (0.36-0.84)	7
Burges 2010[17]	Germany	Europe	100	Mean(range) 60.35(33.12-89.2)	160	IHC	EOC (serous)	Directly	PFS	0.3 (0.13-0.7)	7
Darb-Esfahani 2009[34]	Germany	Europe	139	Mean(range) 57(33-81)	Mean(range) 38(2-118)	IHC	EOC	Directly	OS	0.72 (0.26-1.94)	7
Zamagni 2009[16]	Italy	Europe	35	Mean(range) 67(43-78)	42	PCR	EOC	Indirectly	OS	0.21 (0.05-0.92)	6
Zamagni 2009[16]	Italy	Europe	35	Mean(range) 67(43-78)	42	PCR	EOC	Indirectly	PFS	1.11 (1.01-1.23)	6
Liu 2009[35]	USA	North America	127	NA	100	IHC	EOC (serous)	Indirectly	OS	0.65 (0.41-1.04)	8
Alonso 2009[36]	Spain	Europe	62	Median 56	Median 27	IHC	EOC	Indirectly	OS	9.95 (1.9-51)	8
Alonso 2009[36]	Spain	Europe	62	Median 56	Median 27	IHC	EOC	Indirectly	PFS	1.1 (0.46-2.65)	7
Liu 2010[37]	China	Asia	116	Median(range) 49(30-76)	Median(range) 43(5-93)	IHC	EOC	Directly	OS	1.18 (0.48-2.88)	7
Liu 2010[37]	China	Asia	116	Median(range) 49(30-76)	Median(range) 43(5-93)	IHC	EOC	Directly	PFS	1.16 (0.47-2.86)	7
Schlumbrecht 2011[25]	USA	North America	83	Mean(range) 62.6(34.5-85.9)	Median(range) 38.7(0.5-67.8)	PCR	EOC (serous)	Directly	OS	0.99 (0.94-1.03)	8
Schlumbrecht 2011[25]	USA	North America	83	Mean(range) 62.6(34.5-85.9)	Median(range) 38.7(0.5-67.8)	PCR	EOC (serous)	Directly	RFS	1.02 (0.99-1.04)	8
Halon 2011[18]	Poland	Europe	43	Mean 51	60	IHC	EOC	Indirectly	OS	0.21 (0.05-0.85)	6
Halon 2011[18]	Poland	Europe	43	Mean 51	60	IHC	EOC	Indirectly	PFS	0.47 (0.24-0.95)	6
De Stefano 2011[38]	Italy	Europe	58	Median(range) 54(33-79)	Median(range) 35(9-127)	IHC	EOC (serous)	Directly	OS	0.86 (0.52-1.4)	7
Kolkova 2012[39]	Sweden	Europe	150	NA	120	IHC	EOC	Indirectly	OS	1.08 (0.73-1.6)	7
van Kruchten 2015[19]	Netherlands	Europe	121	Median(range) 61(30-84)	45	IHC	EOC	Directly	OS	1.37 (0.92-2.02)	7
van Kruchten 2015[19]	Netherlands	Europe	121	Median(range) 61(30-84)	45	IHC	EOC	Directly	PFS	1.24 (0.85-1.64)	7
Khandakar 2014[14]	India	Asia	62	Mean 55	70	IHC	EOC (serous)	Indirectly	OS	1.95 (1-3.81)	8
Matsuo 2014[20]	USA	North America	121	Mean(SD) 62.6(10.6)	96	IHC	EOC (serous)	Directly	OS	1.76 (0.7-4.4)	8
Matsuo 2014[20]	USA	North America	121	Mean(SD) 62.6(10.6)	96	IHC	EOC (serous)	Directly	PFS	2.03 (1.01-4.06)	8
Battista 2014[21]	Germany	Europe	108	Mean(range) 61.7(11.4)	Median(range) 43.3(11.4-8)	IHC	EOC	Directly	OS	0.6 (0.28-1.26)	7
Battista 2014[21]	Germany	Europe	108	Mean(range) 61.7(11.4)	Median(range) 43.3(11.4-8)	IHC	EOC	Directly	DFS	0.9 (0.52-1.69)	7
Sieh 2013[7]	USA	Across regions	1691	Mean(range) 60.9(11.4)	Mean(range) 49.2(33.6)	IHC	EOC (serous)	Directly	OS	1 (0.89-1.14)	7
Sieh 2013[7]	USA	Across regions	1691	Mean(range) 60.9(11.4)	Mean(range) 49.2(33.6)	IHC	EOC (serous)	Directly	DFS	1.06 (0.93-1.2)	6
Fujiwara 2012[23]	Japan	Asia	162	Mean(range) 54.1(12.5)	62.4	IHC	EOC	Indirectly	OS	1.31 (0.95-1.81)	6
Aust 2012[24]	Austria	Europe	208	Median(range) 56(18-85)	Median 51	IHC	EOC	Directly	OS	0.51 (0.34-0.77)	6
Aust 2012[24]	Austria	Europe	208	Median(range) 56(18-85)	Median 51	IHC	EOC	Directly	PFS	0.8 (0.58-1.11)	8

EOC: epithelial ovarian cancer; IHC: immunological histological chemistry; PCR: polymerase chain reaction; DCCM: dextran-coated charcoal method; OS: overall survival; PFS: progression-free survival; DFS: disease-free survival; RFS: recurrence-free survival.

### Quality assessment of relationship between ER expression and OS

**Figure 2:**
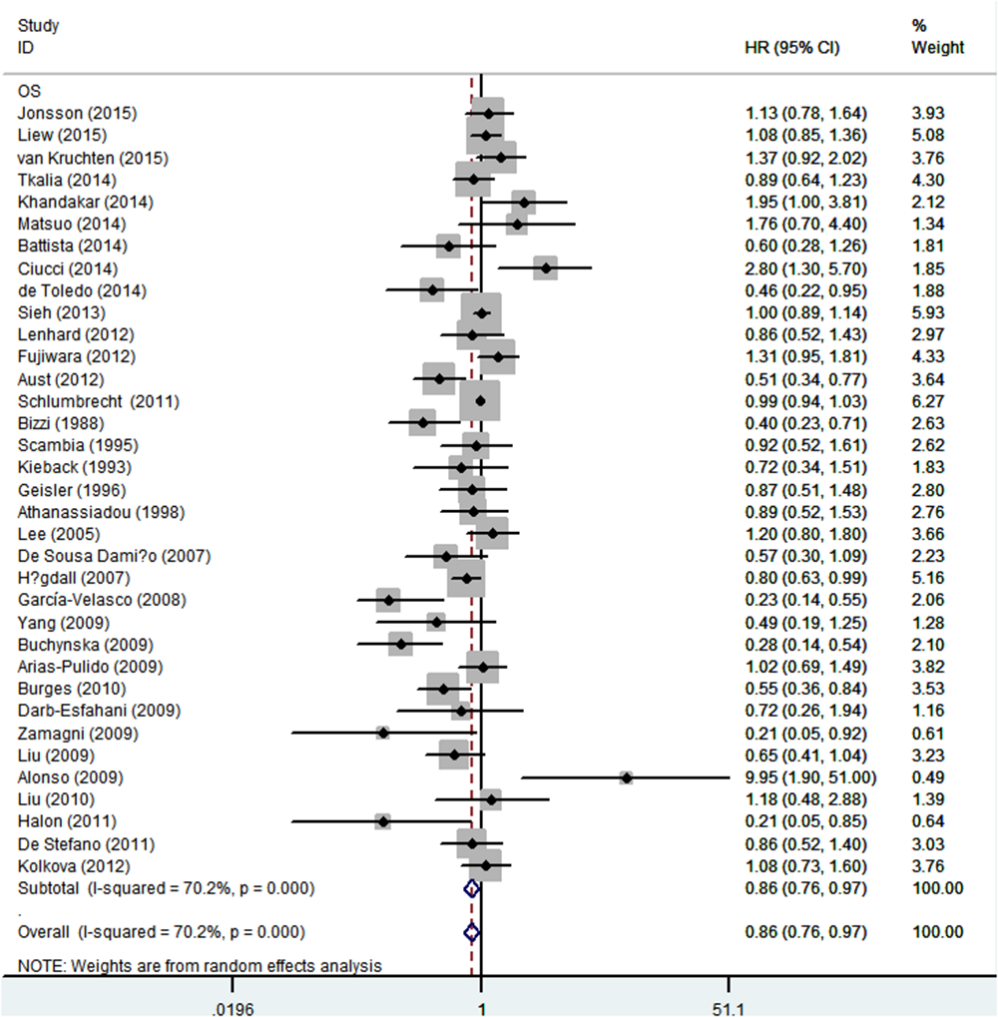
Forest plot of HR and 95% CI in the meta-analysis of the association between estrogen receptor expression and overall survival of ovarian cancer patients Summary of 35 studies, the results showed estrogen receptor was associated with a favorable overall survival of ovarian cancer using random effects model. The % weight was computed automatically by the Stata software.

**Figure 3:**
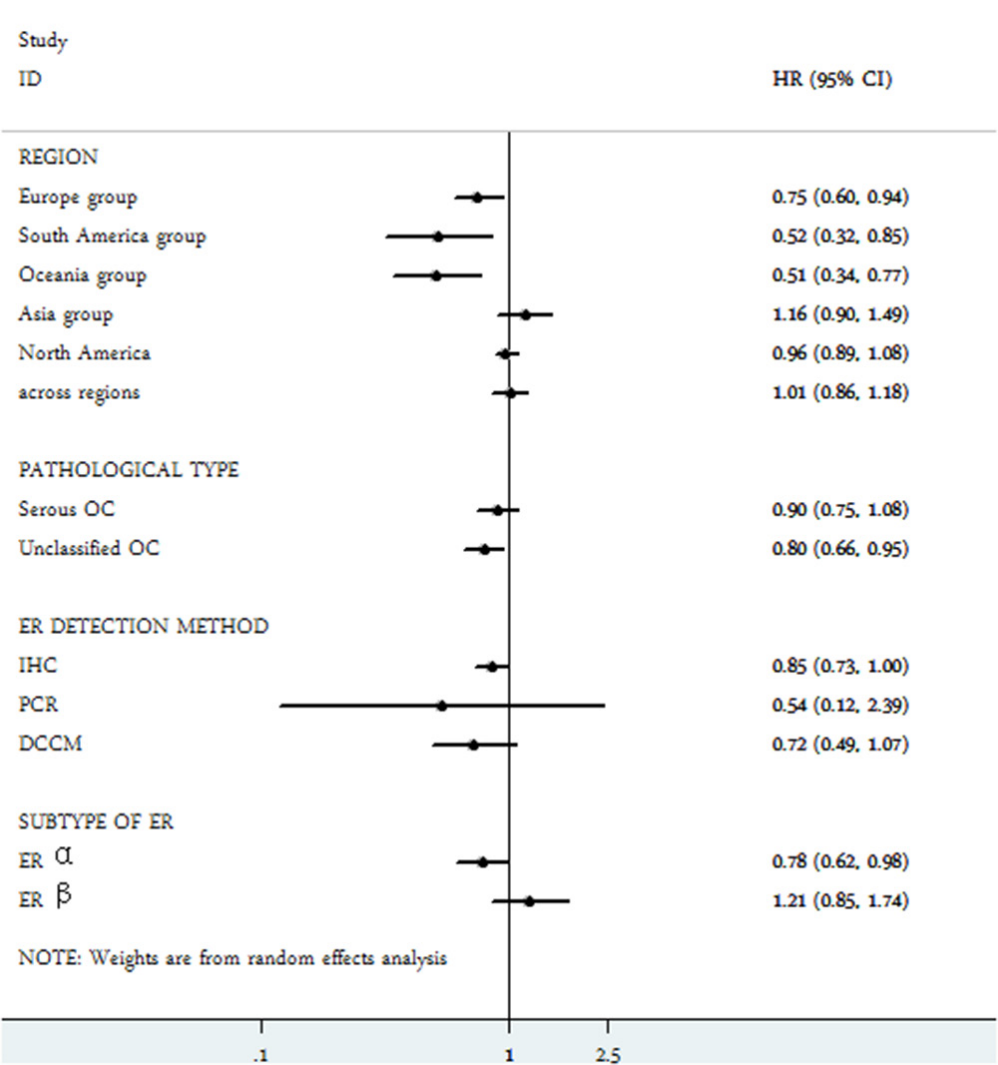
Subgroup analysis of the association between estrogen receptor expression and overall survival of ovarian cancer

A total of 35 studies were included to evaluate the association between ER expression and OS of epithelial ovarian cancer. The pooled data of 35 datasets showed that ER expression was significantly associated with an improved OS of epithelial ovarian cancer (HR = 0.86, 95% CI = 0.76-0.97). A moderate heterogeneity was observed (I^2^ = 70.2%, *P* = 0.00), therefore a random-effect model was used to calculate the pooled HR and 95% CI (Figure 2). Moreover, according to various confounding factors, subgroup analysis was performed to explore the potential source of the heterogeneity among these studies (Figure 3, Table 2).

**Table 2:** Subgroup analysis of the association between estrogen receptor expression and overall survival of ovarian cancer

Subgroup	No. of studies	HR (95%CI)	Heterogeneity	
			*P* value	I^2^(%)
Region				
Europe	19	0.75(0.60,0.94)	0	70.6
South America	2	0.52(0.32,0.85)	0.645	0
Oceania	1	0.51(0.34,0.77)	-	-
Asia	5	1.16(0.90,1.49)	0.228	29.1
North America	7	0.96(0.89,1.08)	0.379	6.3
Across regions	1	1.01(0.86,1.18)	-	-
Pathological type				
Serous OC	12	0.90(0.75,1.08)	0	68.1
Unclassified OC	23	0.80(0.66,0.95)	0	65.8
ER detection method				
IHC	29	0.85(0.73,1.00)	0	67.3
PCR	2	0.54(0.12,2.39)	0.037	77
DCCM	4	0.72(0.49,1.07)	0.127	47.4
Subtype of ER				
ER α	11	0.78(0.62,0.98)	0.005	60.7
ER β	8	1.21(0.85,1.74)	0.017	59

No.: number; HR: hazard ratio; CI: confidence interval; IHC: immunological histological chemistry; PCR: polymerase chain reaction; DCCM: dextran-coated charcoal method.

In the stratified analysis by pathological type, ER expression was associated with a better OS (HR = 0.80, 95% CI = 0.66-0.95) in unclassified epithelial ovarian cancer. Nevertheless, ER expression had no value on overall survival of serous ovarian cancer (HR =0.90, 95% CI = 0.75-1.08).

Subgroup analysis by regions revealed that ER expression was a favorable indicator of OS in South American group (HR = 0.52, 95% CI = 0.32-0.85), Oceanian group (HR = 0.51, 95% CI = 0.34-0.77) and European group (HR = 0.75, 95% CI = 0.60-0.94). Nevertheless, ER positivity was irrelevant to OS of ovarian cancer in Asian population, North American group and the across regions group.

Subgroup analysis based on detection methods for ER expression included immunological histological chemistry (IHC), polymerase chain reaction (PCR) and dextran-coated charcoal method (DCCM). The results suggested that ER expression was related to a favorable OS of epithelial ovarian cancer using IHC for ER detection (HR = 0.85, 95% CI = 0.73-1.00). Nevertheless, when using PCR or DCCM to detect ER expression, no significant correlations were found between ER and OS of epithelial ovarian cancer.

With regard to subtypes of ER α and ER β in epithelial ovarian cancer, as shown in Figure 3, ER α expression (HR = 0.78, 95% CI = 0.62-0.98) had a certain value in predicting a favorable OS, whereas the expression of ER β (HR = 1.21, 95% CI 0.85-1.74) was irrelevant with OS of epithelial ovarian cancer patients.

### Quality assessment of relationship between ER expression and TTP

**Figure 4:**
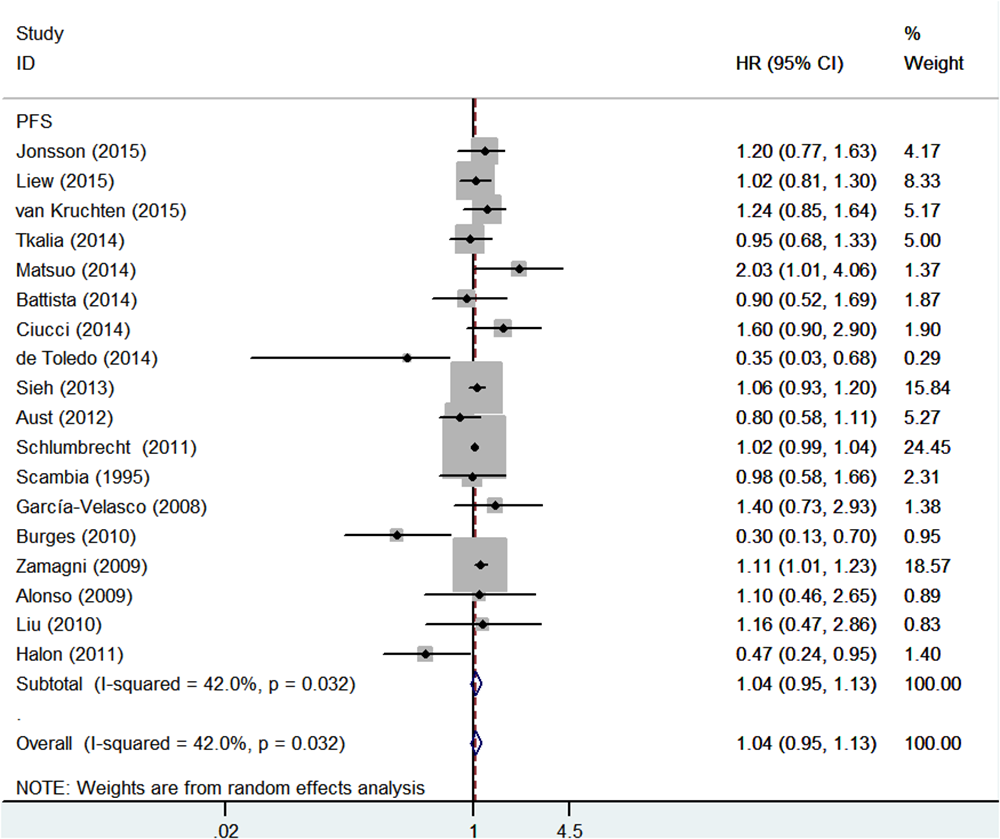
Forest plot of HR and 95% CI in the meta-analysis of the association between estrogen receptor expression and time to tumor progression of ovarian cancer patients Summary of 18 studies, the results showed estrogen receptor was not associated with time to tumor progression of ovarian cancer using random effects model.

**Figure 5:**
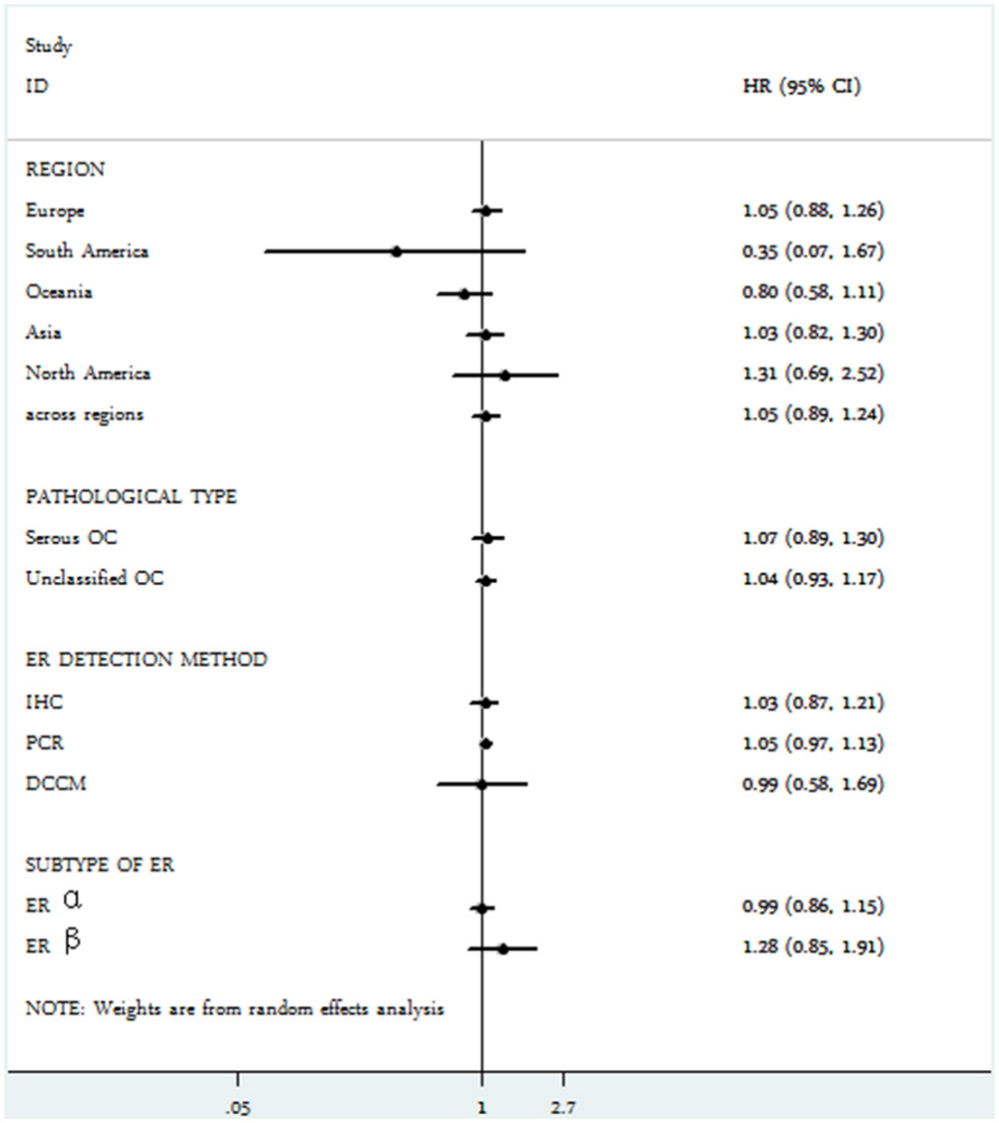
Subgroup analysis of the association between estrogen receptor expression and time to tumor progression of ovarian cancer

Intriguingly, as shown in Figure 4, no significant correlation was observed between ER expression and TTP among epithelial ovarian cancer patients (HR = 1.04, 95% CI = 0.95-1.13)**.** Similarly, neither ER α (HR = 0.99, 95% = CI 0.86-1.15) nor ER β (HR = 1.28, 95% CI = 0.85-1.91) showed effect on TTP of ovarian cancer (Figure 5). Significant heterogeneity was shown among these studies (I^2^ = 42%, *P* = 0.032). Thus, a random-effect model was used for statistical analysis.

We further performed subgroup meta-analysis according to different regions, pathological types, ER detection methods, as expected, ER had no association with TTP of ovarian cancer in all subgroups (Figure 5, Table 3).

**Table 3:** Subgroup analysis of the association between estrogen receptor expression and time to tumor progression of ovarian cancer

Subgroup	No. of studies	HR (95%CI)	Heterogeneity	
			*P* value	I^2^(%)
Region				
Europe	11	1.05(0.88,1.26)	0.048	45.8
South America	1	0.35(0.07,1.67)	-	-
Oceania	1	0.80(0.58,1.11)	-	-
Asia	2	1.03(0.82,1.30)	0.795	0
North America	2	1.31(0.69,2.52)	0.053	73.4
Across regions	1	1.05(0.89,1.24)	-	
Pathological type				
Serous OC	6	1.07(0.89,1.30)	0.012	65.8
Unclassified OC	12	1.04(0.93,1.17)	0.27	17.7
ER detection method				
IHC	15	1.03(0.87,1.21)	0.022	47.1
PCR	2	1.05(0.97,1.13)	0.103	62.4
DCCM	1	0.99(0.58,1.69)	-	-
Subtype of ER				
ER α	7	0.99(0.86,1.15)	0.002	70.9
ER β	4	1.28(0.85,1.91)	0.263	24.7

No.: number; HR: hazard ratio; CI: confidence interval; IHC: immunological histological chemistry; PCR: polymerase chain reaction; DCCM: dextran-coated charcoal method.

### Publication bias

**Figure 6:**
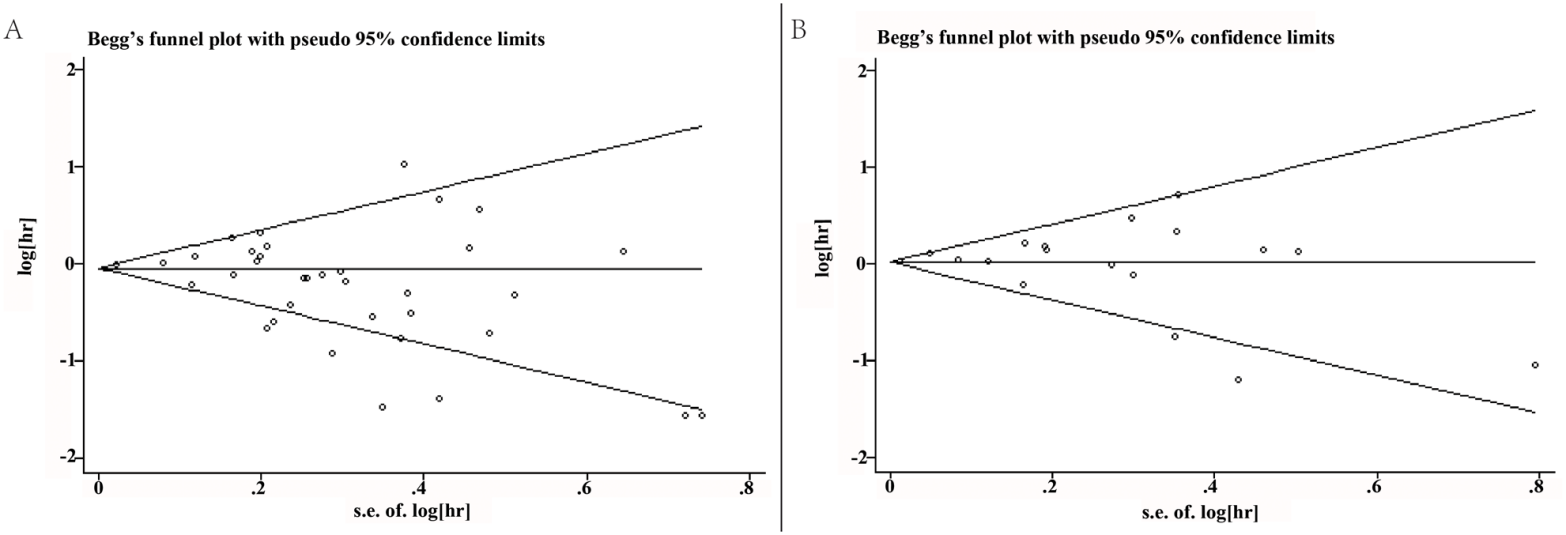
**(A)** Begg's funnel plots for the studies involved in the meta-analysis of estrogen receptor expression and overall survival of ovarian cancer patients. **(B)** Begg's funnel plots for the studies involved in the meta-analysis of estrogen receptor expression and time to tumor progression of ovarian cancer patients. Visual inspection of the Begg’s funnel plot did not indicate substantial asymmetry.

Funnel plots analyses were graphically symmetric, as indicated by Begg’s test, there was no significant publication bias for both OS (Begg’s test, *P* =0.173) and TTP (Begg’s test, *P* = 0.649) among the included studies. (Figure 6).

### Sensitivity analysis

**Figure 7:**
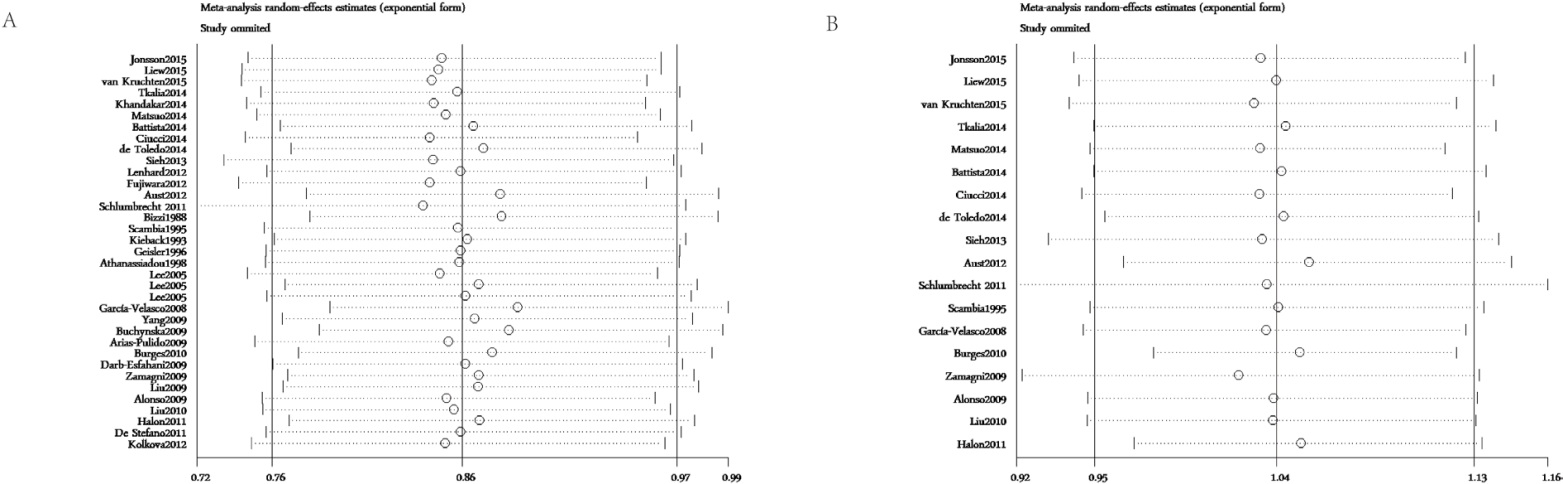
**(A)** Sensitivity analysis of the association between estrogen receptor expression and overall survival in ovarian cancer patients. **(B)** Sensitivity analysis of the association between estrogen receptor expression and time to tumor progression in ovarian cancer patients. The leave-one-out method was used to confirm the stability of the results.

As shown in Figure 7, the leave-one-out method was applied to confirm the stability of the results. Eligible studies were sequentially excluded one by one to evaluate the stability of the obtained conclusions from the remaining data. The statistical significance of the results about OS and TTP was not altered when any single study was omitted. This analysis indicated that the results from this present meta-analysis were reliable.

## DISCUSSION

In the current meta-analysis, we investigated the correlation between ER expression and epithelial ovarian cancer prognosis, and demonstrated that the expression of ER, especially ER α, was a positive predictor of overall survival among epithelial ovarian cancer patients. Nevertheless, ER expression showed no effect on TTP of epithelial ovarian cancer. This study included 35 published articles with a total number of 5824 patients. To our knowledge, this is the most comprehensive assessment of the association between ER expression and epithelial ovarian cancer prognosis to date. Our result is not consistent with the only previous study to explore the prognostic role of ER in ovarian cancer in 2013 [41]. Zhao et al [41] investigated 2784 ovarian cancer patients and found ER failed to predict clinical outcomes of ovarian cancer patients. This can be partly attributed to small sample size. Furthermore, the previous study did not perform subgroup meta-analysis although there was an obvious heterogeneity.

Regarding the histopathological types, our results showed that ER expression was associated with a better OS in unclassified epithelial ovarian cancers, whereas the expression of ER was related to neither OS nor TTP in serous type of cancers. Epithelial ovarian cancers mainly consist of five histological subtypes: high-grade serous cancer, low-grade serous cancer, mucinous, endometrioid, and clear cell cancer [7]. It is now recognized that ovarian cancer subtypes have different etiologies and distinct clinical courses. The association of tumor biomarker expression with survival varies substantially across subtypes [7]. Indeed, the expression of ER differed markedly across ovarian subtypes. Sieh et al [7], in their study on 2933 ovarian cancer patients with various epithelial histology by IHC, reported that the expression of ER was much higher in serous carcinoma and endometriod cancer than in mucinous carcinoma and clear cell carcinoma. Along similar lines, in another large cohort, ER positivity was much lower in the mucinous and clear cell subtype but endometriod type had a similar ER positivity with serous carcinomas [8]. These results suggest that the role and its mechanisms of action of ER in ovary carcinogenesis across ovarian cancer subtypes. Therefore, we suggest that the expression of ER may be a prognostic biomarker in non-serous epithelial ovarian cancer rather than serous ovarian cancer. Further stratification analysis was needed to clarify the prognostic value of ER in each type of non-serous epithelial ovarian cancer.

With respect to source regions, ER expression was correlated with better clinical outcomes of epithelial ovarian patients in European group, South American group and Oceanian group but not in other groups. A probable explanation for these results is that certain genes exert different effects on cancer risk and prognosis across ethnic populations. It is well known that tumor estrogen receptor state differs by race in breast cancer. The proportion of ER-positive tumors was much higher among Non-Hispanic Whites population comparing to Non-Hispanic Black population [42].

Additionally, we studied deeply on the way of ER detection methods. We observed that a positive ER status was generally associated with an improved OS in epithelial ovarian cancer using IHC. Immunohistochemical examination of ER status has been used as a standard-of-care pathological evaluation to guide adjuvant endocrine therapy after surgery of breast cancer [43]. It is the main technology used to determine protein expression status in tissue and has been widely used in the morphological diagnosis of malignancy and contributes to decisions on prognosis. According to our results, we suggested that using immunohistochemistry to evaluate ER expression in postoperative ovarian cancer samples routinely may benefit the prognosis of epithelial ovarian cancer.

Estrogens exert their action through two estrogen receptors (ER α and ER β) [6]. ER α was a well-established biomarker predicting better outcomes in women with breast cancer in Han et al’s study [43]. Consistent with breast cancer, our study demonstrated that ER α predicted a favorable prognosis for ovarian cancer patients. Nevertheless, the expression of ER β was irrelevant with both OS and DFS/RFS/PFS of ovarian cancer patients. A potential mechanism responsible for these findings may be the distinct function of ERs subtypes in the carcinogenesis of the ovary. ER β receptor displays a high expression compared to ER α in normal ovarian epithelium, but this ratio is reversed in ovarian cancers [44].

There are several important implications in this meta-analysis. First, our study showed that ER expression was related to a better outcome of epithelial ovarian cancer, indicating ER may be a potential prognostic biomarker for patients with epithelial ovarian cancer. Second, we identified a subgroup of tumors with unfavorable outcome potentially in epithelial ovarian cancer. Finally, we emphasize the importance of evaluating ER expression by immunohistochemistry in epithelial ovarian cancer paraffin block as a valuable biomarker for prognostic assessment.

Some limitations also existed in this analysis. First, we could only extract summarized population-level data rather than individual patient-level data from the literature. Second, the HR of some studies was indirectly extracted from growth curve, which was less reliable than the data directly obtained from primary literatures. In addition, the heterogeneity across studies could not be eliminated completely, which could result in bias of the outcome. Finally, small studies with negative or null results may not be published, which can cause publication bias. Therefore, further investigations are needed to address the above limitations.

In conclusion, this meta-analysis demonstrates that the expression of ER, especially ER α, is associated with an improved OS, which suggests that ER might be a potential biomarker for prognostic prediction in epithelial ovarian cancer. Additionally, evaluating ER expression by immunohistochemistry in ovarian cancer paraffin is an economical and effective method for predicting ovarian cancer clinical outcomes.

## MATERIALS AND METHODS

### Search strategy

An electronic search of the following databases for relevant studies was performed: PUBMED, EMBASE, and COCHRANE. The research started from November 1980 to December 2016. The search items included “Estrogen receptor” or ER and “Ovarian Neoplasms” or “Ovarian Neoplasm” or “Ovary Neoplasms” or “Ovary Neoplasm” or “Ovary Cancer” or “Ovary Cancers” or “Ovarian Cancer” or “Ovarian Cancers” or “Cancer of Ovary” or “Cancer of the Ovary” and “Prognosis or prognostic or Survival or outcome”. The search was limited to English language articles, but no limitation on regions of publications. Reference lists of all relevant articles were manually screened to ensure the accuracy of the literature search.

### Selection criteria and quality assessment

Studies had to meet the following inclusion criteria: (1) detection of ER expression in primary ovarian cancer tissue; (2) outcomes were survival related, such as overall survival (OS), disease-free survival (DFS), progression-free survival (PFS), or recurrence-free survival (RFS).; (3) original article was written in English.

Exclusion criteria were as follows: (1) review articles, experimental studies, letters, comments, meta-analysis, conference articles or case reports; (2) non-English studies; (3) absence of key information such as hazards ratio (HR), 95% confidence interval (CI) and sample size. Two independent reviewers (ZJS and HL) evaluated eligibility of studies according to the above criteria. The information collected would be repeatedly examined by each other. Disagreements were resolved by discussion.

The Newcastle-Ottawa quality assessment tool was used to estimate the quality of the included studies [45]. We allocated a score of 0-9 to each included article, and those with a score ≥ 6 were considered to be of high quality.

### Data extraction

Data were collected using a predesigned data extraction form by two reviewers (ZJS and HL). We extracted the following data from each studies: first author’s name, year of publication, country of origin, median age, number of patients, pathological type, method of ER assessment, follow-up time, outcome endpoint, HR and 95% CI for ER-positive versus ER-negative.

In most cases, we directly extracted HR and 95% CI from primary studies. If the studies showed inadequate or unclear information, sending an email to the authors for complementary information was our first choice. If the Kaplan-Meier survival curves were available, we used the method as previously described to estimate HR and its corresponding 95% CI [40]. For multiple publications reporting the same study, only the most informative or most recent publication was included in the meta-analysis.

### Statistical analysis

Pooled hazard rate (HR) with its 95% confidential interval (CI) were calculated to measure the prognostic value of ER expression among ovarian cancer patients. Heterogeneity among studies was quantified and assessed using the chi-squared-based Q and I^2^ test and substantial heterogeneity was defined as a *P*-value <0.10. A random-effect model (Der Simonian and Laird method) was used if heterogeneity was observed *P*<0.10, otherwise, the fixed-effect model was used (Mantel-Haenszel method) [46, 47]. Potential publication bias was examined by performing funnel plots qualitatively, and estimated by Begg’s test quantitatively [48, 49]. Sensitivity analyses were employed to find potential origins of heterogeneity and to examine the influence of various exclusions on the combined HR [40]. All analyses were performed using Stata 12.0 software (StataCorp LP, College Station, TX). A *P*-value <0.05 was considered statistically significant.
